# Case Report: Delayed Guillain-Barré syndrome following trauma: A case series and manage considerations

**DOI:** 10.3389/fsurg.2022.903334

**Published:** 2022-08-25

**Authors:** Yiliu Zhang, Chuxin Huang, Wei Lu, Qing Hu

**Affiliations:** Department of Neurology, The Second Xiangya Hospital of Central South University, Changsha, China

**Keywords:** Guillain-Barré syndrome, demyelinating disease, post-trauma, case report, post-surgery

## Abstract

**Aim:**

To analyze clinical associations between Guillain-Barré syndrome (GBS) and trauma.

**Material and Methods:**

We retrospectively reviewed the data of eight patients with post-traumatic GBS between July 2011 and December 2018 at the Second Xiangya Hospital, China, and analyzed the triggers, clinical manifestation, examination results, treatment, prognosis, and potential mechanism related to post-traumatic GBS.

**Results:**

The included patients had GBS preceded by no risk factors other than trauma. Their age ranged from 15 to 60 years (the median age was 52 years), and six patients were males. The potential traumatic triggers included spinal surgery (*n* = 2), high-intensity exercise (*n* = 2), traumatic brain injury (*n* = 1), excessive fatigue (*n* = 1), ischemic stroke (*n* = 1), and cardiopulmonary resuscitation (*n* = 1). The major manifestation was symmetrical limb weakness and/or numbness in all patients. The diagnosis of GBS was based on the results of electromyography, albumino-cytological dissociation, or antiganglioside antibody in cerebrospinal fluid, and other diseases were excluded. Immunotherapy improved symptoms, except in one patient who died.

**Conclusions:**

Trauma is a probable risk factor for GBS that is very easily overlooked, thereby leading to misdiagnosis in clinical practice. We emphasize a new concept of post-traumatic GBS to promote doctors' awareness when they meet people with weakness and sensory deficits after trauma, which benefit early diagnosis, timely treatment, and reduced mortality rate of GBS.

## Introduction

Guillain-Barré syndrome (GBS) is a demyelinating, monophasic polyneuropathy characterized by acute neuromuscular paralysis and albumino-cytological dissociation (AD) in cerebrospinal fluid (CSF). Because two-thirds of patients with GBS manifest flu-like syndrome caused by gastrointestinal and respiratory tract infections before 4 weeks of the onset of weakness, infection is considered a major and important trigger for GBS, particularly Campylobacter jejuni infection (1). In addition, vaccinations have been considered another trigger, although there exist controversial opinions (2). However, in the 1970s, data from Mayo Clinic and Massachusetts General Hospital first recorded 97 cases of postoperative GBS, some of these patients did not involve a history of infection or vaccination, thus, the researchers proposed surgery as a trigger for GBS (3, 4). Furthermore, by presenting a case of GBS that developed after nonoperative traumatic brain injury (TBI), researchers proposed a novel concept, post-traumatic GBS, and defined it as GBS preceded by no risk factors other than trauma (5). In this article, the broadly defined term “trauma” refers to not only physical damage to the body caused by violence or accident, but also the injury caused by any external and internal factors (e.g., surgery).

Herein, we describe eight unique cases of GBS with a history of trauma rather than of infection and vaccination in the past seven years. These cases are collected and analyzed to discuss the possible pathogenesis and clinical characteristics involved in post-traumatic GBS. The report aims to attract more attention to the diagnosis of GBS when patients are presented with acute symptoms of weakness, paresthesia and breathing difficulties after trauma.

## Materials and methods

We retrospectively reviewed the medical records of eight patients who met the established clinical diagnostic criteria of GBS (6) between July 2011 and December 2018 at the Second Xiangya Hospital of Central South University, China. In these patients, GBS developed after surgery- or injury-related trauma. Exclusion criteria included a recent history of respiratory/intestinal infection and vaccination two weeks before GBS onset, a history of administration of monosialotetrahexosyl ganglioside sodium, and other conditions possibly mimicking GBS (6). The following details were collected from the patients' medical records and analyzed: sex, age, types of traumas, the time interval between trauma and onset of GBS, clinical and examination findings, treatment and prognosis. Hughes Functional Grading Scale (HFGS) was used to assess the functional disability of patients with GBS at the onset, the nadir, the time when immunotherapy started, and the discharge [HFGS: 0, normal; 1, minimal signs and symptoms, able to run; 2, able to walk 10 meters unaided, but unable to run; 3, able to walk with aid; 4, bed-bound and not able to lift legs; 5, requiring mechanical ventilation (MV); 6, death (7)]. All patients provided written informed consent before any study-specific procedures and all clinical investigations were conducted under the principles of the Declaration of Helsinki. This study was approved by the Ethics Committee of the Second Xiangya Hospital of Central South University.

## Results

The key characteristics of each case are described below, and the clinical courses are shown in Figure 1, with additional relevant clinical manifestations and laboratory data included in Table 1.

**Figure 1:**
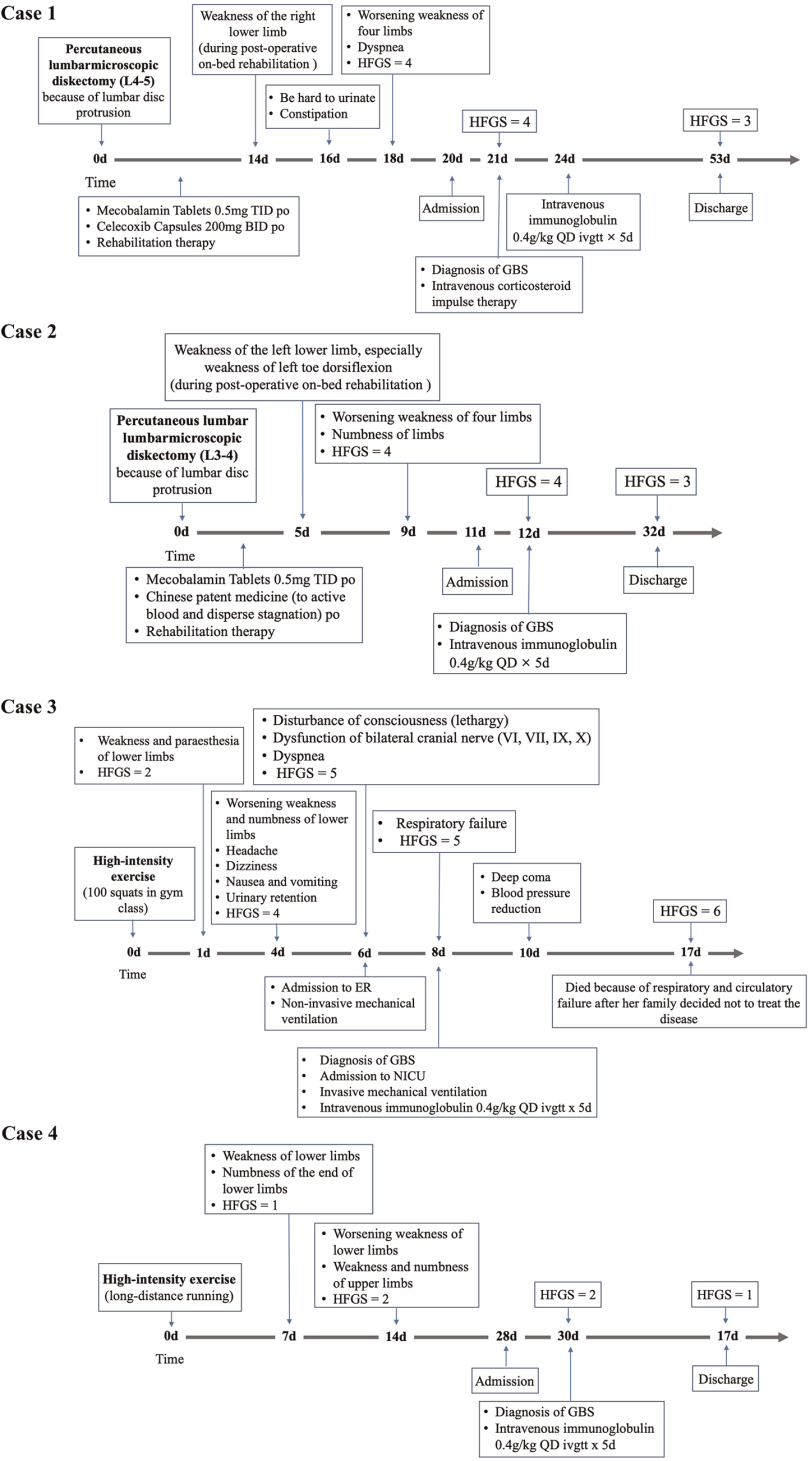


**Figure 1 Continued:**
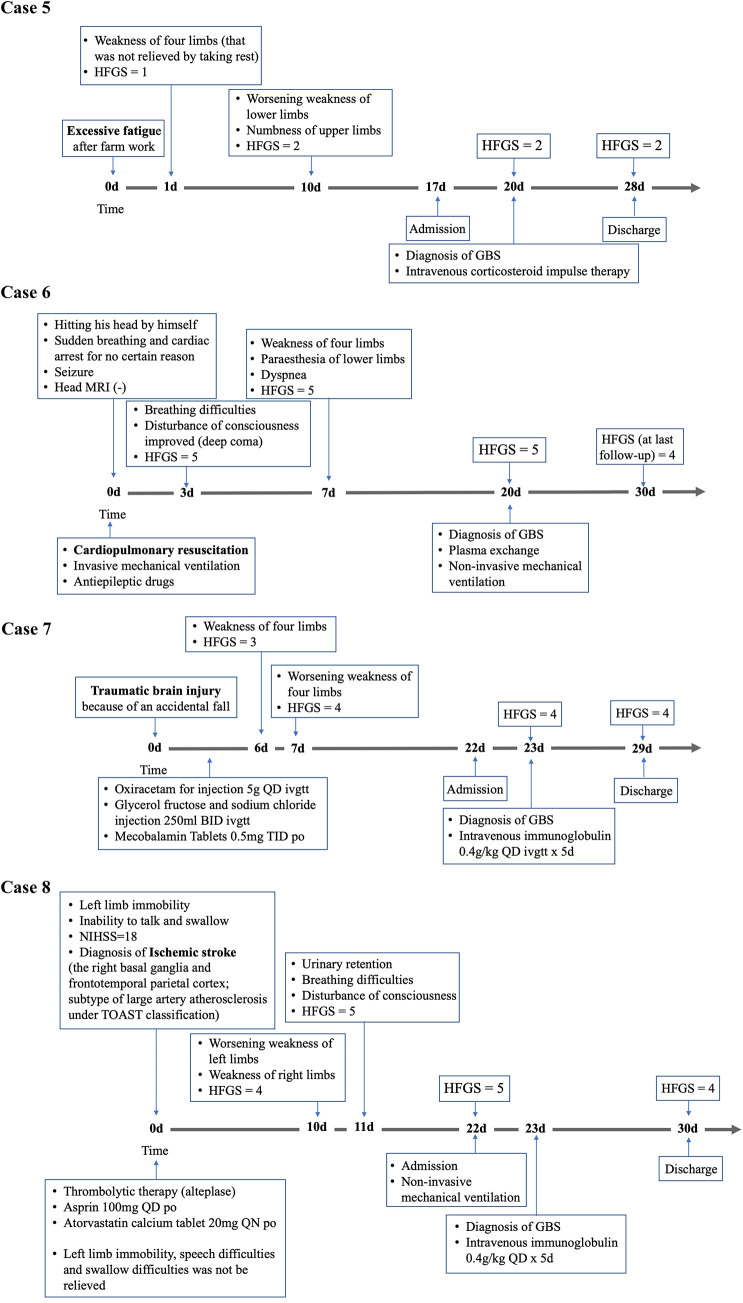
Timeline depicting the significant events in the clinical course of eight patients with GBS

**Table 1:** Clinical manifestations, laboratory data and treatments of eight patients with GBS.

Characteristic	Case 1	Case 2	Case 3	Case 4	Case 5	Case 6	Case 7	Case 8
Age/Sex	58/M	54/F	15/F	18/M	50/M	30/M	60/M	57/M
Etiology of trauma	Spinal surgery	Spinal surgery	High-intensity exercise	High-intensity exercise	Excessive fatigue	CPR	TBI	Ischemic stroke
Time interval (days)								
From trauma to GBS symptom onset	14	5	1	7	1	3	6	10
From GBS onset to nadir	4	4	7	7	9	4	1	1
From GBS onset to admission	6	6	5	14	16	/	16	12
From GBS onset to immuotherapy initiation	7	7	7	16	19	17	17	13
Symptoms at the onset	Limb weakness (RL)	Limb weakness (LL)	Weakness and paraesthesia of lower limbs	Limb weakness and numbness (RL + LL)	Four limbs weakness	Breathing difficulties	Lower limbs weakness	Four limbs weakness
Symptoms at the nadir								
Motor function	Weakness on limbs (RU + LU G3/5, RL G1/5, LL G2/5)	Weakness on limbs (RL G3/5, RU + LU + LL G4/5)	Weakness on limbs (RL + LL G0/5, RU + LU G1/5)	Weakness on limbs (four limbs G4/5)	Weakness on limbs (four limbs G4/5)	Weakness on limbs (RL + LL G2/5, RU + LU G4/5)	Weakness on limbs (RL + LL G2/5, RU + LU G3/5)	Could not cooperate and did not respond to painful stimuli
Deep tendon reflexes	Absent (−)	Absent (−)	Absent (−)	Absent (−)	Decreased (+)	Absent (−)	Absent (−)	Absent (−)
Muscular atrophy	−	+	−	−	−	−	−	+
Cranial nerve dysfunction	−	−	VI, VII, IX, X (bilateral)	−	−	−	−	Could not cooperate
Respiratory muscle involvement	+	−	+	−	−	+	−	+
Objective sensory function	Normal	Hypoesthesia (bilateral forearm)	Hypoesthesia (four limbs)	Hyperesthesia (Below the wrist joints and below the ankle joints of both lower limbs)	Normal	Hyperesthesia (Below the ankle joints of both lower limbs)	Normal	Could not cooperate
EMG	AMAN	ASMAN	NA	AIDP	AMAN	Mixed axonal and demyelinating neuropathy	Severe axonal polyneuropathy	AMAN
Anti-ganglioside antibodies	NA	NA	NA	−	NA	NA	anti-sulfatide Ab in CSF (+); GD1a Ab in serum (+)	NA
CSF laboratory data								
Pressure (mmH_2_O)	NA	NA	119	150	149	123	131	151
Routine test	NA	NA	TCS 2 × 10^6^/L, WBC 1 × 10^6^/L, MNC 1/1	TCS 4 × 10^6^/L, WBC 2 × 10^6^/L, MNC 2/2	TCS 4 × 10^6^/L, WBC 3 × 10^6^/L, MNC 2/3	TCS 2 × 10^6^/L, WBC 2 × 10^6^/L, MNC 2/2	TCS 3 × 10^6^/L, WBC 1 × 10^6^/L, MNC 1/1	TCS 5 × 10^6^/L, WBC 2 × 10^6^/L, MNC 2/2
Biochemical test	NA	NA	Protein 741.60 mg/L ↑, GLU 3.0 mmol/L, chlorides 122 mmol/L	Protein 312.20 mg/L, GLU 2.9 mmol/L, chlorides 126 mmol/L	Protein 2759.70 mg/L ↑, GLU 3.0 mmol/L, chlorides 125 mmol/L	Protein 1795.63 mg/L ↑, GLU 4.0 mmol/L, chlorides 121 mmol/L	Protein 1200.52.mg/L ↑, GLU 2.8 mmol/L, chlorides 123 mmol/L	Protein 1495.66.mg/L ↑, GLU 3.3 mmol/L, chlorides 120 mmol/L
AD	NA	NA	+	−	+	+	+	+
Treatment for GBS	IVIG; HC	IVIG	MV; IVIG	IVIG	HC	MV; PE	IVIG	MV; IVIG
HFGS								
At the nadir	4	4	5	2	2	5	4	5
At the discharge/At the last follow-up	3	3	6	1	2	4	4	4
ΔHFGS	1	1	−1	1	0	1	0	1

NA, not applicable; M, male; F, female; CPR, cardiopulmonary resuscitation; TBI, traumatic brain injury; EMG, electromyography; CSF, cerebrospinal fluid; Ab, antibody; IVIG, intravenous immunoglobulin; PE, plasma exchange; MV, mechanical ventilation; HC, high-dose corticosteroids; AD, albumino-cytological dissociation; AMAN, acute motor axonal neuropathy; ASMAN, acute motor-sensory axonal neuropathy; AIDP, acute inflammatory demyelinating polyneuropathies; RU, right upper limb; LU, left upper limb; RL, right lower limb; LL, left lower limb; TCS, total cellular score; WBC, white blood cell counts; MNC, mononuclear cell; GLU, glucose; HFGS, scores of the hughes functional grading scale, ΔHFGS = (HFGS at the nadir)−(HFGS at the discharge or at the last follow-up).

The age range was 15–60 years (the median age was 52 years), and six patients were males. The traumatic antecedent events of GBS included spinal surgery (*n* = 2), high-intensity exercise (*n* = 2), TBI (*n* = 1), excessive fatigue (*n* = 1), cardiopulmonary resuscitation (CPR; *n* = 1) and ischemic stroke (*n* = 1). Specifically, before the GBS onset, two patients had percutaneous lumbar diskectomy because of lumbar disc protrusion. Another two patients did some exercise (patient 3 did 100 squats, patient 4 had long-distance running) whose intensity was higher than their usual exercise. One patient had excessive fatigue after doing farm work. He complained he felt exhausted, tired and physically weak that could not be relieved by the usual strategies of restoring energy, such as taking rest, which was consistent with the generally accepted definition of fatigue (8). Patient 6 tried to commit suicide by hitting his head by himself. Then he had a seizure in a few minutes. And after one hour, CRP was performed on patient 6 because of sudden breathing and cardiac arrest. The result of his head MRI was negative. Plus, he did not have any symptoms of GBS and pulmonary dysfunction a few days before the CPR. Patient 7 was diagnosed with TBI because of an accidental fall. His CT showed minor intracranial hemorrhage. MRI and cerebral angiography of patient 8 showed ischemic stroke in the right basal ganglia and frontotemporal parietal cortex and 90% stenosis at the initial part of the right internal carotid artery. He was diagnosed with the stroke subtype of large artery atherosclerosis under TOAST classification.

The time interval from trauma to the GBS onset ranged from 1 to 14 days (the median time interval was 5.5 days). The time interval from GBS onset to the nadir ranged from 1 to 9 days (the median time interval was 4 days). However, the median time intervals from GBS onset to discharge and immunotherapy initiations were 12 days and 14.5 days respectively.

All patients did not have any family history of GBS and other peripheral nerve demyelinating diseases. In terms of treatment for the antecedent events, monosialotetrahexosyl ganglioside sodium, the drug for vascular or traumatic central nervous system injury that commonly causes GBS (9), did not be used in any patients. Patient 1 and 2 were treated with non-steroid anti-inflammatory drugs and mecobalamin after spinal surgery. Because of the short time interval from trauma to GBS onset or to the non-specific symptoms of trauma, some patients (#3, #4, #5) did not receive any treatment specifically for their traumatic etiologies. After CPR, Patient 6 was given amiodarone, naloxone and glycerin fructose to support his life and protect brain cells. Patient 7 received symptomatic relief and supportive treatment to decrease intracranial pressure and promote neural regeneration. There was no increase in intracranial hemorrhage during the hospitalization. But he presented with limb weakness after 10 days of TBI. The stroke patient (#8) had thrombolytic therapy with alteplase, but the symptom of left limbs weakness was not improved greatly. After 10 days, he presented with weakness in the right upper and right lower limbs that were not the consequence of stroke progression.

Except the patient 3 who died before completing electromyography (EMG), the EMG of seven patients showed the slowed-down of nerve conduction velocity, decreased wave amplitude, the presence of typical sural sparing pattern, and the prolonged latency of the F wave and H wave. In conclusion, axonal and/or demyelinating damage was recorded in EMG from these patients. AD of CSF was observed in five of eight patients (patient 1 and 2 could not complete lumber puncture because of recent spinal surgery). Tests of anti-ganglioside antibodies in the serum or CSF have been performed in two patients (#4, #7). Only the serum and CSF samples from patient 7 had positive results.

The incidence of respiratory muscle paralysis was high (#1, #3, #6, #8). Patient 3, 6 and 8 received non-invasive or/and invasive MV. The most used treatment was intravenous immunoglobulin (IVIG; *n* = 4). Plasma exchange (PE) and other immunotherapies were also administered to other patients. HFGS at the nadir ranged from 2 to 5 (the median score was 4.5). HFGS at the discharge or the last follow-up ranged from 1 to 6 (the median score was 3.5). Patient 3 who had rapid progress died due to respiratory and circulatory failure during the hospital stay. The median Δ HFGS was 1.

## Discussion

GBS is an immune-mediated inflammatory polyneuropathy that has long been associated with preceding infections and vaccination (1). Recently, there is an emerging relationship between GBS and traumatic events, such as surgery, TBI, bone fracture, chest trauma, abortion and intracerebral hemorrhage (5, 10, 11). But high-intensity exercise, excessive fatigue and CPR triggering GBS are not described before. Here, we refer to “trauma” within the broader context, which includes both external and internal factors leading to all the damage (e.g., surgery, stroke, fatigue, strenuous exercise). Ganglioside displays neurotrophic and neuroprotective properties, and its derivative monosialotetrahexosyl ganglioside sodium are commonly used in patients with vascular or traumatic central nervous system injury (12). GBS is the one of complications of ganglioside (9, 13). So, doctors should be more aware of the diagnosis of GBS when the patients with the history of ganglioside administration after TBI and stroke have weakness, paresthesia and breathing difficulties.

It's reported that post-traumatic GBS is established on the fact that the GBS symptoms start within 30 days of TBI (11) or 6 weeks of undergoing surgery (14), which is in accordance with the clinical records described here (the median time interval was 5.5 days). Most non-traumatic GBS reach the nadir within 2 weeks (15), but the time interval from post-traumatic GBS onset to the nadir in this study ranged from 1 to 9 days which was shorter than non-traumatic GBS. All the patients were presented with motor dysfunction. Some of them were also involved in sensory dysfunction, cranial nerve damage and respiratory muscle paralysis. There is an undeniable fact that motor weakness and sensory dysfunction caused by GBS were symptomatic during the early stage of the traumatic course. However, the GBS symptoms could easily be neglected and misdiagnosed because they are probably confounded by other signs of trauma such as the need for ventilation support, low level of consciousness, multiple organ failure, and other critical illnesses. For example, patient 8 had a low conscious level after an ischemic stroke and did not cooperate with physical examinations. Doctors ignored the worsening limb weakness, then give the patient a delayed diagnosis and treatment of GBS at the time of the first admission. What we should keep in mind is that new symptoms caused by GBS could not be explained by the progression of the primary traumatic events by examination. And physical examinations indicated peripheral neuropathy, including bilateral decreased muscle strength, symmetrical paresthesia, dropped muscle tone, absent deep tendon reflexes and negative pathologic reflex.

Generally, GBS is induced by an abnormal autoimmune response targeting peripheral nerves and their spinal roots (6). The activation of immune cells, in turn, leads to GBS aggravation (16). However, the mechanisms underlying post-traumatic or post-operative GBS remain poorly understood. Post-traumatic GBS is a multifactorial process caused by the post-traumatic changes in the immune system, disruption of the neural system structure and the subsequent activation of the neuroendocrine system. On the one hand, after neurotrauma (4, 17), the blood-brain barrier (BBB) is damaged, which allows the entry of immune cells to clear post-traumatic neuronal debris and take up certain neuronal components as antigens. Thereby, it triggers a chain of immunological events that could further increase the permeability of BBB and the blood-nerve barrier (BNB) (18, 19) and promote autoimmune attacks on peripheral nerves, finally worsening the peripheral nerve demyelination (20). On the other hand, trauma is a stressor activating the hypothalamic-pituitary-adrenal axis (21) and sympathetic nervous system (22), which causes transient immunosuppression (23, 24). This neuroendocrine-mediated turbulence in the immune system enables autoantibodies to attack peripheral nerves. The close association between comorbid autoimmune diseases and post-operative GBS (14) supports that an abnormal immune system has negative effects on GBS development. Besides, during the transient immunosuppression stage, patients are at an increased risk of subclinical and latent infections, which subsequently produce cross-reactive antibodies leading to GBS. What's more, GBS immune susceptibility gene might be related to post-traumatic GBS, such as TLR4 (25) and TNF polymorphism (26). So, we presume that post-traumatic patients with GBS immune susceptibility gene are more prone to GBS. The future studies should examine the difference in promoting-GBS gene between post-traumatic and non-traumatic GBS patients.

CSF analysis and EMG are effective methods for the diagnosis of GBS. Although typical AD (with high protein levels and normal white blood cell count) in GBS is a typical feature of GBS, the normal protein levels in CSF will be found when determined in the first week after the onset of the disease (27). Such as, patient 4 did not have a typical presentation of AD in CSF because a lumbar puncture was performed at an early stage of the disease. Furthermore, various ganglioside antibodies can be detected in the serum of 50% of GBS patients (28). EMG results from all the GBS patients exhibit demyelinating and axonal damage. Compared with non-traumatic GBS, post-traumatic GBS patients were more likely to present with axonal types, such as acute motor axonal neuropathy (AMAN) and acute motor-sensory axonal neuropathy (ASMAN). It may be the reason why post-traumatic GBS would have a severe clinical course and subsequent poor prognosis, which is coincident with previous studies (11, 29). Because of the limited sample size in existing literature, future research is needed to confirm whether post-traumatic GBS patients are inclined to have a certain subtype.

What's more, acquired paralysis is a common and severe complication in one-third of critically ill patients in the intensive care unit (ICU), including critical illness polyneuropathy, critical illness myopathy and critical illness polyneuromyopathy. They are caused by sepsis, multiple organ failure, and systemic inflammatory response syndrome (30). Meanwhile, these causes have also been encountered by GBS patients in ICU (31). Despite some similarities between acquired paralysis and GBS in terms of clinical manifestation, they could be distinct in terms of the features of CSF results, EMG, antiganglioside antibody and creatine kinase in serum and neuromuscular histopathology (30). Therefore, GBS could be a neurological complication after trauma. Clinicians should pay attention to GBS diagnosis and differentiate post-traumatic GBS from acquired paralysis when encountering patients with persistent weakness.

First-line treatments for GBS include PE alone and IVIG alone. However, in some areas of China, due to the limitation of financial conditions and medical care, glucocorticoids are used for some patients who cannot afford standard PE and IVIG treatments (#1, #5). Approximately 20%–30% of patients have respiratory insufficiency and need MV during the progressive phase which could influence negatively the GBS outcomes (15), while our study suggests that the patients with post-traumatic GBS probably have a higher chance to develop respiratory muscle paralysis (#1, #3, #6, #8). Thus, patients with suspected post-traumatic GBS should be monitored for respiratory failure and have timely ventilation support. Even though HFGS at the nadir ranged from 2 to 5, we found Δ HFGS ranged from −1 to 1, which indicates the patients with post-traumatic have poorer outcomes because of delayed diagnosis and inappropriate treatment. Therefore, treatment for GBS still should be started as soon as possible before the irreversible nerve damage and poor outcomes (32).

There is another significant fact about the relationship between trauma and GBS that we cannot neglect. Some factors, such as blood glucose, base excess, mean arterial pressure, PaO2/FiO2 ratio and serum hemoglobin, are closely associated with the mortality of patients after trauma (33). It's reported that these factors were poor prognostic index of GBS (34). Various scores including similar items are available to evaluate GBS outcomes (35, 36). In other words, disease management after trauma and GBS has a lot in common. Therefore, establishing the association and concept of post-traumatic GBS not only further helps decrease the trauma-related mortality rate, but also improves the quality of life and long-term outcomes of patients who had GBS after trauma.

Given this is a small case series from a clinical center in Changsha, China, it is undeniable that we cannot provide a concrete causal relationship and clear mechanism behind it. Secondly, since our cases are retrospective and some GBS symptoms might be confounded by traumatic symptoms in the clinical course, the time of GBS onset is not very accurate. However, we exclude the other confounding factors, including the drug use causing GBS and preceding infection history through their laboratory examinations before the GBS onset, such as blood routine test, urine routine test, stool routine test, C respond protein and chest CT. We provide a possible association of GBS with traumatic events. Despite these limitations, it is notable that the clinical presentations and laboratory findings in these patients appear consistent with previous published single case reports of GBS after surgery or TBI. And more large-scale research on the association between GBS and trauma is needed.

## Conclusions

Based on this retrospective analysis of eight patients and in combination with literature reviews, we presume that various types of traumas are important triggers for GBS. Usually, these triggers are more likely to be overlooked and misdiagnosed, thereby causing the poor prognosis for patients with GBS. Additionally, it's worth emphasizing the management and evaluation of patients with trauma since early predictive factors on mortality of traumatic patients can also decrease the number of preventable deaths and minimize GBS-related disabilities. Therefore, we believe that it is necessary to raise the awareness of clinicians about post-traumatic GBS, which can lead to early and precise diagnosis and treatment of GBS among patients of post-trauma.

## Data Availability

The datasets presented in this article are not readily available because of ethical/privacy restrictions. Requests to access the datasets should be directed to the corresponding author.
